# Xerostomia after Radiotherapy for Oral and Oropharyngeal Cancer: Increasing Salivary Flow with Tasteless Sugar-free Chewing Gum

**DOI:** 10.3389/fonc.2016.00111

**Published:** 2016-05-03

**Authors:** Julie Killerup Kaae, Lone Stenfeldt, Jesper Grau Eriksen

**Affiliations:** ^1^Department of Oncology, Odense University Hospital, Odense, Denmark

**Keywords:** xerostomia, chewing gum, radiotherapy, oral cancer, oropharyngeal cancer

## Abstract

**Introduction:**

Radiation-induced xerostomia is a frequent late side effect after treatment for oral and oropharyngeal cancers. This may induce swallowing difficulties, compromised oral well-being, reduced nutrition intake, or speech deficiencies. Consequently, quality of life is often impaired for these patients.

**Objectives:**

The purpose of this study was to investigate the possibility to mechanically stimulate residual saliva function by using tasteless and sugar-free chewing gum. It was hypothesized that tasteless and sugar-free chewing gum could immediately increase salivary flow and potentially improve oral well-being when used on a regular basis.

**Methods:**

From October to December 2014, 31 consecutive patients treated with primary radiotherapy (RT) and concomitant cisplatin (in locally advanced cases) for oral or oropharyngeal cancer consented to participate. All patients had finalized RT 2–8 months prior to participation and suffered from xerostomia. Samples of unstimulated and chewing gum-stimulated saliva were obtained at the entry into the study (Visit 1). For 2 weeks, patients used chewing gum on a regular basis whereupon saliva measurements were repeated to verify the changes (Visit 2). An abbreviated EORTC H&N35 questionnaire was completed for both visits. A small control group consisting of young and healthy individuals also tested the chewing gum.

**Results:**

Twenty patients completed the study and an increase in saliva flow was observed for 14 patients. Before and after intervention with chewing gum, an increase in mean saliva output was seen between unstimulated and stimulated saliva for both Visit 1 and 2 (*p* = 0.008 and *p* = 0.05, respectively). No change in saliva output was seen in the control group.

**Conclusion:**

The chewing gum was able to stimulate saliva output that was seen at the beginning and at the end of the intervention. No improvement in baseline saliva was seen. Relevant changes in subjective measures of xerostomia were seen after 2 weeks of chewing the gum.

## Introduction

Xerostomia is a common acute and late side effect when treating head and neck cancer (HNC) patients with curative intended radiotherapy (RT) (1–3). Xerostomia is the subjective feeling of oral dryness, whereas hyposalivation is the physiological reduction in salivary flow (4). Hyposalivation is defined as unstimulated whole saliva flow of ≤0.2 mL/min, and symptoms of xerostomia often become evident when saliva flow is below 0.1–0.2 mL/min (5, 6). Xerostomia is not necessarily correlated to a reduction in saliva flow and may also be present independent of hyposalivation. Healthy individuals produce between 0.5 and 1.5 L of saliva daily, with saliva being secreted by the three paired major salivary glands. It consists of approximately 99% water and 1% proteins and salt, and normal daily secretion of saliva is vital for maintaining good oral health, nutritional intake, and communication skills (7, 8).

The severity of xerostomia caused by RT is affected by the total dose and dose per fraction and is often irreversible (9, 10). To assess the severity of xerostomia in the clinic, a good approach is to make use of an observer-based scoring system, and a validated quality of life measurement device and measuring salivary flow (11). Various treatments are available to relieve the discomfort of xerostomia, including symptomatic relief by oral lubricants and saliva substitutes. Salivary stimulants may be considered where residual salivary gland function remains (12).

The purpose of this study was to investigate the possibility to mechanically stimulate residual saliva function. It was hypothesized that by using a tasteless and sugar-free chewing gum, an immediate increase in saliva flow would be obtained and potentially improve oral well-being when used on a regular basis.

## Materials and Methods

This non-randomized cohort study was conducted at the Department of Oncology at Odense University Hospital from October to December 2014. HNC patients diagnosed with oral or oropharyngeal carcinomas were eligible for participation after completing either curative intended treatment with intensity-modulated radiation therapy (IMRT) or postoperative IMRT with or without concomitant cisplatin.

Patients were recruited during follow-up visits to the department 2–12 months after having completed treatment. Furthermore, all patients had to suffer from xerostomia (slight/moderate/severe) as graded by the attending physician according to the Danish Head and Neck cancer Group (DAHANCA) follow-up guidelines. Medical records for all consenting patients were reviewed with regard to medication-induced xerostomia.

For each patient, the study consisted of two visits at the department separated by 14 days. All oral intakes 1 h before onset of the test, which potentially could influence the test results, were not recommended. Visit 1 consisted of four elements:

(1) An unstimulated saliva sample.
(2) An abbreviated version of the EORTC H&N35 questionnaire.
(3) Testing the chewing gum.
(4) A stimulated saliva sample.

First, the unstimulated saliva sample was obtained by the patient spitting into a test tube for 5 min. No sipping of water or saliva stimulant was allowed 1 h before and during the collection of the saliva sample. Second, an abbreviated version of EORTC H&N35 questionnaire (including only the oral and food-related questions plus two additional questions added for the purpose of the study) (Table 2) was completed to asses xerostomia grade, quality of life, and difficulties associated with xerostomia (13, 14). Afterward, a saliva stimulant in the form of a tasteless sugar-free chewing gum was tested and chewed for 5 min supported by a metronome (60 beats/min). After depositing the chewing gum, the stimulated saliva sample was obtained by the patient spitting into a second test tube for 5 min.

The study chewing gum was distributed for use at home in-between visits, and patients were instructed to use it three to five times a day including before regular meals.

At Visit 2, the patient repeated all tests conducted during Visit 1. A second unstimulated and stimulated saliva sample was obtained, and the EORTC H&N35-abbreviated questionnaire was completed again.

### Chewing Gum

For this study, a specially designed chewing gum to stimulate whole saliva flow was used. The chewing gum contained neither taste nor sugar, had no hard coating, and consisted of a basic gum base added mannitol. The chewing gum conformed to the relevant European Union legislations on foods. For practical use, it was reduced in size to accommodate any inconvenience for the recruited patients dealing with pain in the mouth or having trouble overcoming large food objects.

### Control Group

To compare and evaluate the saliva stimulating effects of the chewing gum, the product was tested on a small control group (*n* = 10) consisting of young, healthy, non-smoking, and non-medicated students/health-care professionals. None reported problems regarding xerostomia. All participants were instructed in the spitting and chewing procedures and provided saliva samples. One unstimulated and stimulated saliva sample was obtained from each participant.

### Saliva Measurement

Saliva samples were weighted and the salivary flow rate was calculated in grams per minute (7). For all sample tubes pre- and postweight were measured on a Mettler Toledo (Colombus, OH, USA) weight. To determine the exact weight of saliva, the weight was calibrated with 500, 100, and 1 g weights before use. After weighting, all samples were centrifuged at 2000 × *g* and 20°C for 5 min before the volume was determined by use of 3 mL pipettes. For this study, the weight of the saliva output was considered most accurate.

### Ethics

Written informed consent was obtained from all patients agreeing to participate. The study was approved by the Regional Scientific Ethical Committes for Southern Denmark. The study was conducted and the data analyzed without involvement from Fertin Pharma who produced the chewing gum.

### Statistics

The primary endpoint was the immediate increase in saliva output after stimulation with tasteless sugar-free chewing gum at Visit 1. The secondary endpoints included oral well-being estimated by the abbreviated EORTC H&N35 questionnaire.

Saliva output was expected to be normally distributed. The correlations between measurements of the saliva output were tested using a paired *t*-test. Patient and tumor characteristics for all eligible patients and responses to the questionnaires were tested by Spearman’s correlation for categorical values. A two-sided *p*-value <0.05 was regarded as significant. Data were analyzed using SPSS version 22 for Windows.

## Results

A total of 62 consecutive HNC patients were assessed for eligibility. Thirty-one patients declined participation. Among the 31 included patients, 11 patients did not complete due to problems with prosthetic teeth (*n* = 2), distance to the hospital (*n* = 2), withdrawal of consent without explanation (*n* = 5), or screening failure (*n* = 2). In total, 20 patients completed the study and underwent saliva collection 4 times while testing the chewing gum during the 2-week study period.

The study group consisted of 15 men and 5 women, age ranging from 46 to 73 years (median 62 years) and all current non-smokers. Considering the follow-up period after RT, 10 patients had completed treatment within 2–5 months, whereas 10 patients had finished within 6–12 months. The patients declining participation did not differ from the group of patients consenting to participation with regard to gender, age, follow-up after RT, tumor site, clinical stage, or distance to hospital. Only smoking tended to be more prominent among patients who did not enter the study (*p* = 0.01) (Table 1).

**Table 1:** **Patient and tumor characteristics for all eligible patients**.

	Total^a^	Non-participants^b^	Participants^c^	*p*	Study group^d^	*p*^e^

	*n* = 62	*n* = 31	*n* = 31		*n* = 20	
Men	42 (68%)	18 (58%)	24 (77%)	NS	15 (75%)	NS
Age (median) (range, years)	63 (39–78)	64 (39–78)	62 (46–73)	NS	62 (46–73)	NS
Smoking after RT	10 (16%)	9 (29%)	1 (3%)	0.01	0	NS
Follow-up after RT						
2–5 months	34 (55%)	18 (58%)	16 (52%)	NS	10 (50%)	NS
6–9 months	13 (21%)	7 (23%)	6 (19%)		5 (25%)	
10–12 months	14 (23%)	7 (23%)	7 (23%)		5 (25%)	
>12 months	1 (2%)		1 (3%)			
Site						
Pharynx	48 (77%)	25 (81%)	23 (79%)	NS	15 (75%)	NS
Oral cavity	10 (16%)	5 (16%)	5 (16%)		3 (15%)	
Saliva gland	1 (2%)	1 (3%)		-	
Unknown primary tumor	3 (5%)		1 (3%)	2 (7%)		2 (10%)	
Clinical stage III–IV	51 (82%)	27 (87%)	24 (77%)	NS	16 (80%)	NS
Concomitant chemotherapy	32 (52%)	13 (31%)	19 (61%)	NS	10 (50%)	0.05
Distance to hospital						
>50 km	24 (39%)	12 (36%)	12 (41%)	NS	10 (50%)	NS
<50 km	38 (61%)	20 (65%)	18 (58%)		10 (50%)	

*^a^Total number of consecutive and eligible patients asked to participated in the study*.

*^b^Number of patients declining to participate including screening failure*.

*^c^Number of patients consenting to participate including patients not completing Visit 2*.

*^d^Number of patients with repeating measurements who complete the study*.

*^e^*p*-Value comparing the patients completing the study with the patients lost to follow up*.

*RT, radiotherapy; NS, no significant *p*-value*.

### Xerostomia

All study patients reported xerostomia before testing the chewing gum. Responses to the abbreviated EORTC H&N35 questionnaire at Visit 1 showed that xerostomia was a major complaint, with 90% of the patients rating xerostomia as “quite a bit” or “a lot” (Table 2). At Visit 2, only 30% of the patients rated xerostomia as “quite a bit,” and none experienced complaints corresponding to “a lot.” No significant difference was found for the patient-reported evaluations of xerostomia. Medical records revealed that five patients were prescribed opioids, antidepressants, and antiepileptic medication, and all five patients had pronounced xerostomia complaints (“quite a bite” or “a lot”).

**Table 2:** **Responses to the abbreviated EORTC H&N35 questionnaire from participants completing the study (*n* = 20)**.

		Visit 1				Visit 2				*p*^a^

In the past week have you had…		None	A little	Quite a bit	A lot	None	A little	Quite a bit	A lot	
**Oral cavity**										
Q1	Pain in your mouth?	13 (65%)	4 (20%)	2 (10%)	1 (5%)	17 (85%)	2 (10%)	1 (5%)		0.05
Q2	Pain in your jaw?	14 (70%)	4 (20%)	1 (5%)	1 (5%)	18 (90%)	1 (5%)	1 (5%)		0.01
Q4	A dry mouth?	1 (5%)	1 (5%)	8 (40%)	10 (50%)	7 (35%)	7 (35%)	6 (30%)		NS
Q5	Sticky saliva?	4 (20%)	8 (40%)	5 (25%)	3 (15%)	5 (25%)	7 (35%)	7 (35%)	1 (5%)	NS
Q6	Less saliva?	7 (35%)	2 (10%)	5 (25%)	6 (30%)	13 (65%)	3 (15%)	3 (15%)	1 (5%)	0.007
**Eating difficulties**										
Q7	Problems swallowing liquids?	13 (65%)	6 (30%)	1 (5%)		15 (75%)	4 (20%)	1 (5%)		NS
Q8^X^	Problems swallowing solid food?	7 (35%)	5 (25%)	4 (20%)	3 (15%)	6 (30%)	8 (40%)	5 (25%)	1 (5%)	NS
Q11	Decreased sense of taste?	8 (40%)	4 (20%)	3 (15%)	5 (25%)	12 (60%)	4 (20%)	3 (15%)	1 (5%)	0.01
Q13	Trouble enjoying your meals?	5 (25%)	3 (15%)	7 (35%)	5 (25%)	6 (30%)	9 (45%)	4 (20%)	1 (5%)	0.004
Q14	Trouble eating with other people?	12 (60%)	4 (20%)	3 (15%)	1 (5%)	10 (50%)	6 (30%)	4 (20%)		NS
Q19	Increased saliva flow after using the chewing gum?	–	–	–	–	“Yes” 19 (95%)	“No” 1 (5%)			NS

*Q6 and Q19 are added questions*.

*^a^The *p*-value was found by using a paired *T*-test*.

*Q is the number of the question in the questionnaire*.

*^X^Q8: one response is missing from questionnaire 1*.

*NS is no significant *p*-value*.

### Oral Complaints

Pain in the mouth or jaw and eating difficulties were also evaluated with the abbreviated EORTC H&N35 questionnaire before and after intervention with the chewing gum (Table 2) and was significantly reduced after 2 weeks of intervention (*p* = 0.05 and *p* = 0.01, respectively). Furthermore, an increase was reported in the subjective feeling of total amount of saliva in the mouth (*p* = 0.007). In a total of 19 patients, 95% reported a subjective increase in saliva flow after intervention with the chewing gum. Eating difficulties in terms of swallowing issues were not found to differ significantly, whereas patients reported less trouble enjoying their meals after Visit 2 (*p* = 0.004).

### Salivary Output

**Figure 1:**
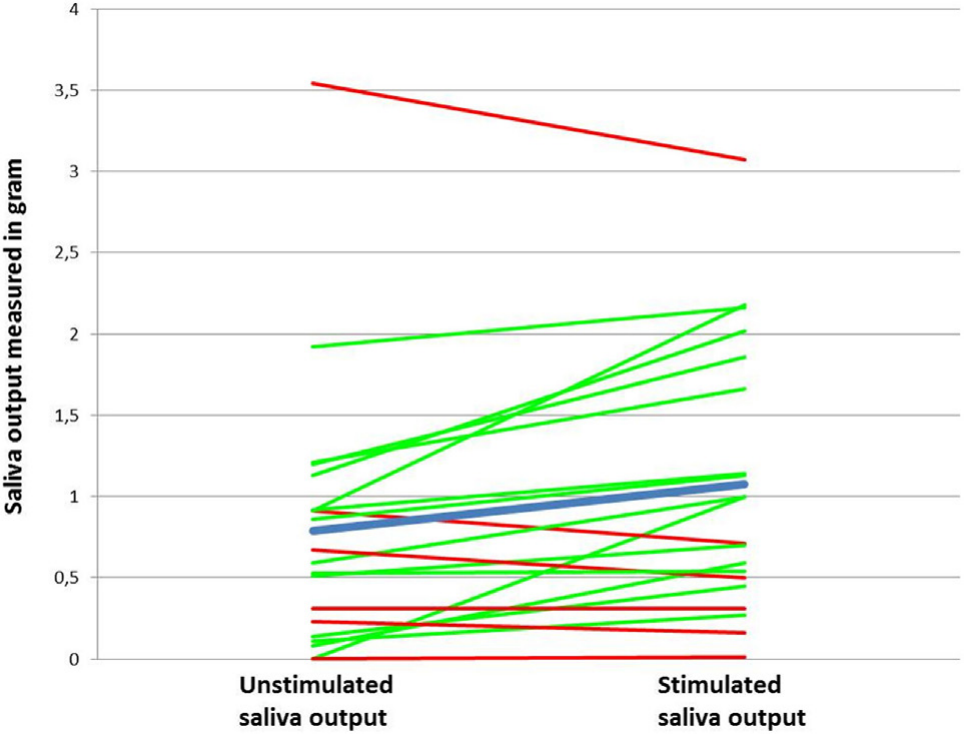
**Increase and decrease in saliva output after stimulation with the chewing gum (*n* = 20) from Visit 1**. The green line represents an increase in saliva output after stimulation. The red line represents a decrease or no change. The blue line represents the mean value.

**Figure 2:**
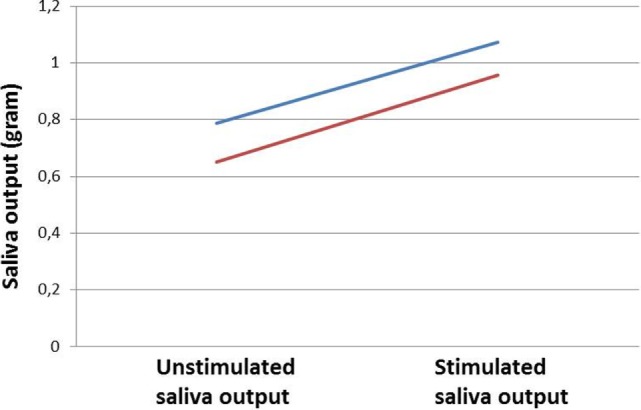
**Mean output for unstimulated and stimulated saliva measured in grams**. The blue line represents saliva output at Visit 1 and the red line at Visit 2.

Mean distribution and individual data of saliva output for patients at Visit 1 are illustrated in Figure 1. At Visit 1, the mean unstimulated saliva output and stimulated saliva output were 0.79 and 1.07 g versus 0.62 and 0.82 g at Visit 2 after intervention with the chewing gum (Figure 2). The increase in saliva output was found to be significant for both Visit 1 and 2 when tested by the two-sided *t*-test (*p* = 0.008 and *p* = 0.05, respectively). No significant difference in stimulated output between Visits 1 and 2 was found (*p* = 0.2). When testing mean saliva flow rate in grams per minute (*p* = 0.05 and *p* = 0.04) and saliva volume (*p* = 0.02 and *p* = 0.05), the increase in saliva was also found to be significant.

The mean flow rate for unstimulated saliva was 0.09 mL/min after using chewing gum for 2 weeks. The definition for very low unstimulated whole saliva flow rate is <0.1 mL/min and corresponds with hyposalivation (6). Looking at all measurements of the unstimulated flow rate (milliliters per minute), nine patients had a very low flow rate below 0.1 mL/min, and seven patients had a low flow rate between 0.1 and 0.2 mL/min.

Saliva output for patients prescribed with xerostomia-induced medication (*n* = 5) did not seem to affect the results of chewing gum intervention, with mean unstimulated and stimulated saliva output being 1.25 and 1.52 g at Visit 1.

Comparing the mean saliva output from the study group at Visit 1 and Visit 2 with the mean saliva output from the control group, an increase in saliva production was not seen in the control group. For the control group, both mean unstimulated and stimulated saliva output were much higher than the results presented in the study group (4.01 and 3.96 g, respectively). No significant difference was found between mean unstimulated and stimulated saliva output for the control group.

## Discussion

In this study, HNC patients treated with curative intended RT and concomitant chemotherapy had at least 0.2 g increase in saliva output after intervention with the tasteless and sugar-free chewing gum. For both Visit 1 and Visit 2, saliva output increased when stimulated by the chewing gum (*p* = 0.008 and *p* = 0.05, respectively). This corresponded well with the patients reporting a positive subjective change regarding their xerostomia complaint. No special parotid sparing techniques were used for the patients included in the study. However, dose constraints to the parotid glands [*D*_mean_ ≤ 20 Gy (contralateral) or *D*_mean_ ≤ 26 Gy (both)], submandibular glands (*D*_mean_ ≤ 35 Gy), and oral cavity (*D*_mean_ ≤ 30 Gy) were intended to be met in every single case.

With xerostomia being both an early and late side effect to primary curative intended RT for oral and oropharyngeal carcinomas, it is common that many patients will experience a permanent subjective feeling of dry mouth. Xerostomia is not a life-threatening complication; however, decreased saliva flow and changes in saliva composition are straining. Saliva often become sticky and unmanageable, which compromises oral well-being and results in eating difficulties, increased risk of caries, and impaired speaking abilities. Living with xerostomia may inflict emotional strain such as worry, tension, and depression and limit social activities (10, 12, 15).

Various salivary substitutes and stimulants are on the market to relieve discomfort associated with xerostomia. Substitutes, such as sprays, gels, mouthwashes, and special toothpaste, are developed to keep soft tissue moist and increase the viscosity of liquids (16). Substitutes are limited by their short duration of effect, often unpleasant taste, and high cost but can be useful during the night (17). The saliva stimulants include sugar-free chewing gum, ascorbic acid (vitamin C tablets), malic acid, and pilocarpine (18). There is low level of evidence concerning the efficiency of the various products available.

For HNC patients, it remains a challenge to find suitable products to increase salivary flow. After ending RT, the oral cavity is highly sensitive due to decreased salivary flow rate and alterations to the sense of taste. Strong flavors, such as peppermint or lemon, are not favored in the early phase of recovery (19). Sugar may contribute to or worsen xerostomia and increases the risk of carries. The chewing muscles can be restrained due to radiation sequelae, and some patients are confined to processing small food items.

The chewing gum designed for this study tries to accommodate the need of the HNC patient. The lack of taste serves the purpose of stimulating the salivary flow by physical and mechanical stimulation alone, whereas the soft texture and small size makes it easy to chew regardless of rigid muscles or oral sensitivity. This is in agreement with others suggesting that saliva stimulants in the form of a sugar-free chewing gum can aid to promote salivation when residual salivary glandular function remains (20). The gum increases salivary flow by stimulating the taste receptors and through physical stimulation of the salivary gland (12, 18, 21).

When comparing mean unstimulated and stimulated saliva output for both Visit 1 and Visit 2, a relative similar increase in salivary output was seen at both visits (Figure 2). The study was not able to find an absolute increase in whole unstimulated saliva output after the second visit, indicating that 2 weeks of regular use of chewing gum did not increase unstimulated saliva output. The decline may be explained by the short period of time allotted for the patient to adapt to the chewing gum, failure to use it on a regular basis, or progressing xerostomia after RT. Furthermore, the study from Flink et al. argues that the circadian rhythm and fasting may also have a negative impact on the saliva secretion in patients with hyposalivation (6). In this study, measurement of saliva flow took place throughout the day time with no regard to the circadian rhythm of saliva secretion. Due to scheduling difficulties, it was not possible to conduct Visit 2 at the exact same time slot or location for all study patients.

Despite including 31 eligible HNC patients, only 20 patients completed the study. After completing treatment for HNC, the patients are only frequenting the department for scheduled checkups with a span of 3–6 months. Participating in this study required an extra visit to the hospital for saliva collection and evaluation of the chewing gum alone. Some of the patients lost to follow-up did not allow time for an extra visit due to long travel distances or coincide with work.

The study did not look into changes in the saliva composition, and further studies should include testing the variations of saliva composition. The chewing gum used in this study did not include any lubricating or fluoride additives. Further testing ought to include chewing gum with emollient additives in order to see whether it is possible to make changes in the saliva composition and further improve the oral well-being.

## Conclusion

Using chewing gum as a saliva stimulant, the study was able to stimulate and increase mean salivary output for 14 out of 20 consecutive HNC patients. The majority of the patients reported a self-rated improvement regarding xerostomia complaints and improved oral well-being after being subjected to the study chewing gum.

## Author Contributions

All the three authors have been actively involved in the design of the study, analyzing of the data, and writing the manuscript. Dr. JK and Dr. JE have been actively involved in the collection of data.

## Conflict of Interest Statement

The authors declare that the research was conducted in the absence of any commercial or financial relationships that could be construed as a potential conflict of interest.
